#  Assessment of Response to Treatment in Patients with Otomycosis

**Published:** 2018-01

**Authors:** Keyvan Kiakojori, Nasim Bagherpour Jamnani, Soraya Khafri, Saeid Mahdavi Omran

**Affiliations:** 1 *Department* * of Otorhinolaryngology* *, * *Faculty of Medicine, * *Babol University of Medical Sciences, Babol, Iran.*; 2 *Student, Faculty of Medicine, Babol University of Medical Sciences, Babol, Iran.*; 3 *Department of Biostatistics, School of Medicine,* * Babol University of Medical Sciences, Babol, Iran.*; 4 *Infectious Diseases & Tropical Medicine Research Center, Health Research Center, Faculty of Medicine, Babol University of Medical Sciences, Babol, Iran. *

**Keywords:** External ear, Fungal infection, Otomycosis, Recurrence rate

## Abstract

**Introduction:**

Due to the prevalence of otomycosis in the north of Iran, which has a humid climate, this study aimed to examine the recurrence rate of otomycosis in Iranian patients.

**Materials and Methods:**

This cross-sectional study was performed in all patients suspected of otomycosis referred to the Otorhinolaryngology Clinic of Ayatollah Rouhani Hospital of Babol. Demographic and clinical data of patients as well as their symptoms were collected in a checklist at referral time and also 14 days after referral.

**Results:**

The results showed that 11 (7.3%) cases had a recurrence of otomycosis. There was no significant relationship between the incidence of otomycosis and age or sex (P=0.86 and 0.88, respectively). Septate mycelium was the most observed element on direct examination. Aspergillus flavus was the most common pathogenic agent in culture media, followed by Aspergillus niger and Candida albicans.

**Conclusion:**

Based on the results of this research in Babol, the recurrence of otomycosis was 7.3% and was related to swelling and erythema in ear canal.

## Introduction

The term, ‘otomycosis’ is mainly used to describe external fungal infections affecting areas such as the auricle, auditory duct, eardrum, and middle ear (1). A fungal infection of the ear is a chronic, acute or sub-acute inflammation of the ear which rarely involves the middle ear (2). Species that cause fungal infections in the ear include saprophytic filamentous fungi, yeast and dermatophytes. Aspergillus and Candida species are the most common fungi in otomycosis (1,3-6). This infection is usually symptomatic, and its main symptoms include itching, pain, tingling, and loss of hearing. Clinical examination usually shows white, gray, and black or cheese-like tissues and inflammation on the outer ear (7-10). Otomycosis is usually a secondary infection, which can be related to various risk factors such as swimming, hot weather, the absence of cerumen, working in a dry and dusty environment, use of hearing aids, poor health status, genetic factors and ear surgery (2,11-14). Otomycosis is prevalent among adults, while other age groups such as children may also be affected. It has a higher prevalence in the 21–30-year age group, and lower among individuals 10 years or younger and over 60 years of age (2,11). Interestingly, some articles have reported a higher prevalence of the disease among women, while others have reported a higher prevalence among men (2,15).

The rate of recurrence of otomycosis in a report published by Jia et al. in China was 8.98% among 108 patients, and it was reported that recurrence was not uncommon, and that it is difficult to eradicate the infection in patients with diabetes and mastoid cavity (16). In another study conducted in Nigeria in 5,784 patients with ear diseases, 378 cases (54.6%) had otomycosis, among whom the recurrence rate was reported for 17 patients after 6 months of treatment (4.50%) (3).

The recommended treatment for otomycosis involves local debridement, local and systemic antifungals, and the discontinuation of local antimicrobials (15,17). This infection is rarely life-threatening, and local antifungals such as clotrimazole, miconazole and nystatin are essentially effective against otomycosis (2,18,19). Local clotrimazole is the most common antifungal used in such treatments (16,20). Due to the prevalence of otomycosis and its recurrence resulting from Iran's climate, this study aimed to examine the recurrence rate for otomycosis in these patients.

## Materials and Methods

*Participants*

This cross-sectional study was performed in all patients suspected of otomycosis referred to the Otorhinolaryngology Clinic of Ayatollah Rouhani Hospital of Babol. Inclusion criteria included patients who had otitis externa, and the exclusion criteria included patients who had no fungal infection on direct examination and culture, or only had bacterial cells or had a fungal and bacterial combination (pathogenic bacteria based on the cultivation results), and those who were suffering from perforations. Patients with no follow up or a non-response to calls were excluded from this research.

The patient's profile included demographic information, previous history of ear illness and treatments, ear manipulation, having contact with moisture (such as swimming in a pool or the sea), and symptoms at the time of visit, as well as symptoms after 14 days. This information, along with the exact time of experiencing symptom relief after treatment, were collected in a checklist.

*Examinations*

Portions of samples were placed directly on the slide for direct examination. These slides were fixed after drying by gentle heat and then stained with methylene blue. Other portions of the samples were cultured on two plates of Sabouraud agar supplemented chloramphenicol (Sc) at 25°C and 37°C for 2 weeks, and one plate of Sabouraud agar containing chloramphenicol and cycloheximide (Scc) at 25°C for 4 weeks. Finally, another portion of the samples was cultured for further assurance and the examination of both fungal and bacterial infection in chocolate agar (anaerobic place) and blood agar medium. These plates were kept at 37° C for a maximum of 48 hours.

In the case of a positive result on direct examination for the presence of fungal elements including mycelia (with or without septate), reproductive organs, including conidiophore, vesicle, phialide and conidia, or pseudo mycelium and negative results for bacteriology, the patient was considered to have otomycosis. In this case, routine treatments were initiated, and the patient was evaluated for recurrence rate. A slide culture test (for filamentous fungi) and culture on the Chromogenic CHROMagar Candida medium and the production of a germ tube and vesicles on the corn meal agar (for yeast) were performed to identify those fungi at the genus or species level, if necessary.

*Treatment*

If patients referred with perichondritis, severe inflammation, redness of the canal and the presence of humidity, an initial antibacterial antibiotic such as a ciprofloxacin tablet, alongside an analgesic and anti-inflammation agents, was given due to canal obstruction and the impossibility of clinical examination and local treatment. After relief of inflammation, local treatment in the form of clearance suction and removal of colonies was performed; sometimes 2% oxygenate solution or distilled water or normal saline serum were used, and the area was dried. After examining the eardrum and ensuring non-perforation, 2% Miconazole ointment was applied in the canal. After eradication of clinical symptoms, patients were considered to be successfully treated cases. The researcher was blind to each treatment in patients.

*Recurrence assay*

The evaluation period for recurrence assay was 3 months. The patient was evaluated by an ear, nose and throat (ENT) specialist 2 weeks after treatment, based on the questionnaire form. 

The patient was asked to contact their doctor by telephone or meet in hospital within 3 months if they encountered a problem. 

After 3 months, the patient was also be referred to an ENT specialist in the hospital for a recurrence assay. This situation may occur after the referral of the patient due to the return of problems in the ear canal according to physician observation.

*Statistical analysis*

Data were analyzed using SPSS V.22 software. Fisher’s exact test was used for qualitative variables. In other cases, frequency and percentage were used. P-value less than 0.05 were considered significant.

## Results

In total, 205 patients were examined within 1 year, of which, six cases were excluded due to eardrum perforation in the initial examination, and 49 were excluded based on the results of culture (observing only bacterial infection or bacterial and fungal infection). Overall, 150 patients were finally included in the study. The mean age of the participants was 44.55±19.24 years (minimum age, 4.5 months; maximum, age 89 years). 

Demographic information showed that the prevalence of otomycosis was relatively equal in both genders (50.67% in male patients and 49.33% in female patients). The most commonly affected occupation was employees who carry out office work (59.33%), followed by housekeepers (26.67%) and the self employed (1.33%). The place of residence was urban for most patients (72%), and 28% of patients came from a village.

Among patients, 129(86%) cases reported ear manipulation; 67(44.7%) patients were swimmers, and a previous history of external ear disease was expressed in 100(66.7%) patients. The reasons for visiting the ENT clinic was hearing loss in 149 patients (99.3%). The most common reason for the first visit was hearing loss followed by secretion (80.7%), pain (75.3%) and itching (76%). On the second visit, 138(92%) patients had no complaint about the hearing loss problem, and swelling and secretion had decreased (Table.1).

**Table 1:** Causes of hospital visit in otomycosis patients referred to Ayatollah Rouhani Hospital

**Variables**	**First visit,** **Number (%)**	**Second visit,** **Number (%)**
Pain	113(75.33)	12(8)
Swelling	95(63.33)	10(6.67)
Itching	114(76)	6 (4)
Secretion	121(80.67)	10(6.67)
Hearing loss	149(99.33)	12(8)
Infected ear		
Left	69(46)	5(3.33)
Right	81(54)	7(4.67)

Swelling and inflammation decreased from 76.7% to 3.3% between the first and second visit. The ear canal was normal in 90% of patients. The color of secretion mostly was white and cheesy (46.7%) (Table.2).

**Table 2:** Symptoms of patients with otomycosis diseases in their first and second visit

**Variables**	**Symptoms**	
	**First visit Number (%)**	**Second visit Number (%)**
**Ear canal condition**		
Healthy	1(0.67)	140(93.33)
Swollen and inflamed	115(76.67)	5(3.33)
Swollen and wounded	31(20.67)	5(3.33)
Swollen and narrow canal	3(2)	0(0)
**Eardrum**		
Healthy, no rupture	135(90)	147(98)
No rupture, but wounded	15(10)	3(2)
**Properties of secretion**		
No secretion	0(0)	140(93.33)
White and brown	21(14)	1(0.67)
White and cheesy	70(46.67)	4(2.67)
Black and brown	25(16.67)	2(1.33)
Black and Cheesy	23(15.33)	3(2)
black and white	11(7.33)	1(0.67)
**Involved ear**		
Right	70(46.67)	8(5.33)
Left	80(53.33)	3(2)
		

**Fig 1:**
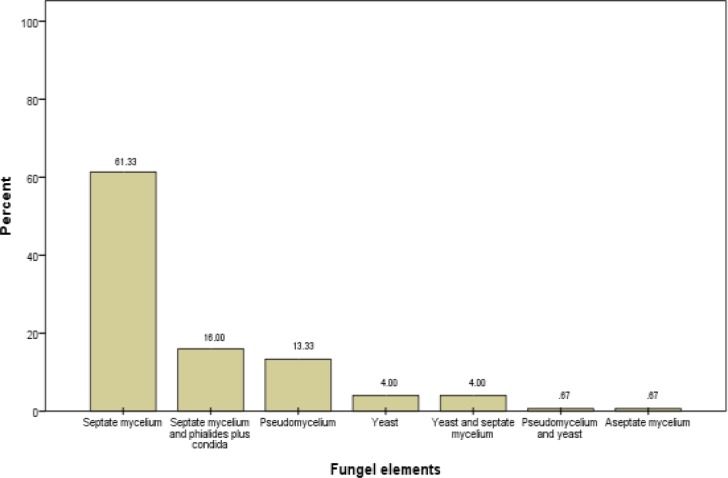
Frequency of fungal distribution elements in otomycosis

On microscopic examination, several fungal elements were observed, of which the most commonly observed element was septate branched mycelium and then pseudo mycelium and yeast. In some cases, fruiting bodies such as conidiophores, vesicles, phialide and conidia were observed (Fig.1).

In terms of the prevalence of fungal species identified by cultivation, A. flavus was the most common pathogenic species with the highest frequency (33.33%), followed by A. niger (30%) and Candida (12.66%). Aspergillus spp. was the most common in all genuses in this study (79.33%). More than one fungus as etiologic agents were found in a few cases (Table. 3).

**Table 3:** Frequency of fungal species in samples obtained from otomycosis according to gender

**Fungi**	**Male**	**Female**	**Total** Number
A. nige	20 (26.31)	25 (33.78)	45 (30)
A. flavus	26 (34.21)	24 (32.43)	50 (33.33)
A. fumigatus	3 (3.95)	6 (8.11)	9 (6)
A. Terreus	3 (3.95)	4 (5.41)	7 (4.67)
Aspergillus sp.	5 (6.58)	3 (4.06)	8 (5.33)
Candida spp.	11 (14.47)	8 (10.81)	19 (12.66)
Cladosporium spp.	1 (1.32)	0 (0)	1 (0.67)
Yeast	4 (5.26)	1 (1.35)	5 (3.34)
Rhizopus spp.	0 (0)	1 (1.35)	1 (0.67)
A. niger + yeast	2 (2.63)	0 (0)	2 (1.33)
A. niger + Candida	0 (0)	1 (1.35)	1 (0.67)
A. flavus + yeast	1 (1.32)	1 (1.35)	2 (1.33)
Total Number	76 (100)	74 (100)	150 (100)
			

Overall, 11 cases (7.33%) had a recurrence of symptoms, of whom four patients (2.67%) were less than 30 years of age. The frequency distribution of otomycosis recurrence in the different age groups showed that there was no significant relationship between otomycosis recurrence and age group (P=0.88). The recurrence of disease was slightly higher in women (4%) than in men (3.33%), but there was no significant relationship with gender (P=0.76). All recurrent cases were Aspergillus genus. Fungi isolated in patients with recurrent otomycosis were A. niger (4%), A. flavus (1.33%), A. fumigatus, A. terreus and Candida spp. (0.67% each).

Swollen and inflamed or wounded in ear canal with recurrence of otomycosis was resolved only in one patient, and was still present in 10 patients with a swollen and 

inflamed or wounded ear canal on the second visit. Patients with swollen, inflamed and wounded tympanic membrane showed recurrence of otomycosis on the second visit in comparison with the normal ear canal (P<0.001) (Table.4).

**Table 4:** Recurrence in the ear canal and tympanic membrane at the first and second visits

**Variables**	**No Number (%)**	**Yes Number (%)**	P-value
**Condition of ear canal at first visit**			0.001
Healthy	0(0)	1(0.67)	
Swollen and inflamed	3(2)	112(74.67)	
Swollen and wounded	8(5.33)	23(15.33)	
Swollen and narrow canal	0(0)	3(2)	
**Tympanic condition at first visit**			0.08
Healthy	8(5.33)	127(84.67)	
No rapture, no wound	3 (2)	12 (8)	
**Condition of ear canal at 2** ^nd^ ** visit**			<0.001
Healthy	1(0.67)	139(92.67)	
Swollen and inflamed	5(3.33)	0 (0)	
Swollen and wounded	5(3.33)	0 (0)	
Swollen and narrow canal	0(0)	0 (0)	
**Tympanic condition at 2** ^nd^ ** visit**			<0.001
Healthy	8(5.33)	139(92.67)	
No rapture, no wound	3(2)	0(0)	

## Discussion

The main aim of this study was to examine the recurrence of otomycosis in patients who referred to Ayatollah Rouhani Hospital; the results indicated that the recurrence rate was 7.33%. Further, the recurrence of disease was not related to the age and gender of the patients, although it was related to the condition of the ear canal (swollen, inflammation and wound) in the second visit.

The study of otomycosis is important for two reasons; first, ENT specialists have problems with the treatment of otomycosis, and second, patients are severely affected for months from such a chronic illness (21). In this study, the frequency of otomycosis was not different between the two genders, but the disease was more common among those who work outside the house. In contrast, Afshari et al. indicated that the frequency of otomycosis was higher in males than in females. This difference can be due to differences in geographical location and demographic characteristics, because Afshari studied the military and their families (22), while we studied ordinary members of society. Furthermore, the prevalence of otomycosis may vary in different age groups in the two genders. For example, Kiakojouri et al. reported that the incidence of externa otitis in male children was higher than in females (23). This could be due to the greater playfulness of boys or a lack of hygiene among boys during their childhood. Nemati and Kiakojouri concluded that the prevalence of otomycosis among women was higher than in men (24,25). Jia et al. found that this ratio was 2 to 1 for females to males (16). Other studies indicated opposite results, suggesting that men are at increased risk of otomycosis (1,26). This controversy and these contradictory results suggest the need for more studies to be conducted in this area.

Employees were the mostly affected, possibly due to the fact that opportunistic and pathogenic fungi are abundant in the environment and are transmitted to human ears through dust and contaminated equipment. However, it would be expected that this issue would affect the self-employed occupation to a greater extent, because they are in contact with more contaminated equipment and environment because of their job requirements. It seems that this hypothesis therefore requires further investigation and more comprehensive studies.

A total of 86% of patients reported in this study had ear manipulation. Loh et al. also found that manipulation and cleansing of the ear could be an important factor in exacerbating the disease in many cases (27). Hearing loss was the most common and important cause of patient referral, followed by secretion, itching and pain, which was consistent with the results of the study by Kazemi et al. in this regard. They found that the feeling of itching in the ear canal, ear cramps, loss of auditory power, pain, and possibly the observation of secretion in the ear canal were the causes of patients referring to hospital (28).

In this study, the A. flavus fungus was the most common pathogen of otomycosis among patients. However, in studies conducted by Beaney et al., Kaur et al. and Pahwa et al., the main fungal agents were the Aspergillus and Candida species (27,29,30). This finding is in contrast with the results of the present study in some conditions. It should be noted that a wide range of fungi are known as causative agents for otomycosis, but since this disease is mostly observed in tropical and subtropical regions of the world, it is expected that in each region a special genus of fungi is the most common cause of otomycosis due to the climatic conditions and individual lifestyles. Kiakojouri et al. also studied patients with otomycosis in 2015. They found that A. niger was the most abundant species causing otomycosis (25).

According to the results of the present study, among the 11 patients who had recurrence, some of them had swollen and wounded or inflamed ear canal on the first or second visits. It seems that an inflamed and wounded ear canal is one of the most effective factors for predicting the recurrence of otomycosis. In 2012, Abou-halawa et al. conducted a study in 40 patients, of whom five had recurrence (12.5%) which was related to factors such as appropriate and timely treatment, full use of medications, and the level of ear infection (31). Jia et al. reported a recurrence rate of 8.8%, higher than that of the present study (16). This different rate in recurrence of otomycosis may be due to social and economic differences. In 2008, Fasunla et al. reported a recurrence rate of 4.5% after 6 months of treatment (3). Although this level was less than that of the present study, the living and climate conditions in different regions, as well as the type and duration of treatment, were factors affecting the recurrence, which were completely different in the two studies. In the present study, Aspergillus spp. was the most commonly present fungus in patients with recurrence of otomycosis. Ozcan et al. (2003) commented that the effects of fungal species on the recurrence of otomycosis is one of the key issues to be addressed (32), and this study is one of the few studies to have examined this issue.

In this study, the recurrence rate was higher in individuals aged less than 30 years, but age is not a risk factor for recurrence of disease. The obtained recurrence rate can be dependent on a variety of factors such as the level of hygiene, having different hobbies such as swimming, a dusty working environment, and having the habit of ear manipulation.

Comparison of the results of this study with previous studies indicates that there are differences between studies due to the definite effects of climatic and geographical conditions, social hygiene and the types of occupations of patients and occupational risk factors in different societies.

## Conclusion

According to the results of this study, the otomycosis recurrence rate in Babol was 7.3%, and the most common fungal species related in these patients was *A. niger*. The lack of evaluation of the risk factors affecting the recurrence of otomycosis is one of the limitations of this research; thus it is recommended that the incidence of otomycosis in different geographical areas and the factors influencing its recurrence are studied. Due to the importance of keeping the ear dry, it is suggested that, while receiving treatment, the patient should protect the ear canal from moisture.
